# Persistence of acidosis in alloxan-induced diabetic rats treated with the juice of *Asystasia gangetica* leaves

**DOI:** 10.4103/0973-1296.75887

**Published:** 2011

**Authors:** Solomon O. Rotimi, Omolola E. Omotosho, Oluwakemi A. Rotimi

**Affiliations:** *Biochemistry Unit, Department of Biological Sciences, Covenant University, Ota, Nigeria*; 1 *Department of Biochemistry, University of Agriculture, Abeokuta, Ogun State, Nigeria*

**Keywords:** Acidosis, *Asystasia gangetica*, hypolipideamic, hypoglycaemic

## Abstract

**Background:**

Diabetes mellitus is gradually becoming a global health burden leading to an increase in the search for herbal hypoglycemic agents as alternatives to synthetic ones. ***Asystasia gangetica*** is one of the herbs used in folklore system of medicine for managing hypoglycaemia associated with diabetes.

**Materials and Methods:**

The influence of the juice of ***A. gangetica*** leaf on alloxan-induced diabetic rats was assessed by treating diabetic rats with 25%, 50% and 75% fresh juice and glibenclamide for 5 weeks. Afterwards, the plasma concentrations of glucose, triacylglycerols, total cholesterol, high-density lipoprotein (HDL) cholesterol, thiobarbituric acid reactive substances and bicarbonate were assayed spectrophotometrically.

**Results:**

Treatment of the diabetic rats with the juice significantly (*P* < 0.05) reduced the elevated plasma levels of glucose to a level not significantly (*P* > 0.05) different from that of glibenclamide. The juice also significantly (*P* < 0.05) reduced the plasma lipid peroxidation and improved the lipid profile, as indicated by a significant (*P* < 0.05) reduction in the total cholesterol: HDL cholesterol ratio. However, there was a significant (*P* < 0.05) rise in the level of bicarbonate as result of the juice treatment from 28.15 ± 2.82 mmol/l in normal control to 60.83 ± 17.46 mmol/l in diabetic control and to 122.20 ± 34.68 mmol/l, 120.95 ± 35.09 mmol/l and 115.85 ± 11.79 mmol/l in 25%, 50% and 75% juice, respectively.

**Conclusion:**

Therefore, this inability of ***A. gangetica*** to prevent acidosis detracts from the potential of its usefulness in managing diabetes.

## INTRODUCTION

Diabetes mellitus is a group of syndrome characterized by hyperglycemia, altered metabolism of lipids, carbohydrates and proteins associated with an increased risk of complications from vascular disease.[1] The prevalence of diabetes is on the increase globally and in African communities due to the ageing of the population and drastic lifestyle changes accompanying urbanization and westernization.[2] Also, studies from five West African communities in Nigeria and Ghana have identified genes within populations that create susceptibility to diabetes.[3] Hence, it represents a growing burden of health care systems of African countries, most of which already face difficult economic conditions.

Diabetes is a chronic disease; hence, a scrupulous control is needed to help reduce hyperglycemia and the risk of long-term complications, which are known to be the major causes of morbidity and mortality. Therefore, a holistic therapy for diabetes should go beyond reversing hyperglycemia and dyslipidemia associated with diabetes.[4]

Since time immemorial, patients with diabetes mellitus have been treated orally by folklore with a variety of plant extracts.[5] Nowadays, more than 1,200 species of plant (from 725 genera belonging to 183 families) are used to treat symptoms of diabetes mellitus. One of these traditional herbs that is used to manage hyperglycemia is *Asystasia gangetica* (L) T. Anderson, sub-specie micrantha (Nees) Ensermu (Acanthaceae).[6,7]

*A. gangetica* is an attractive, fast growing, spreading, herbaceous ground cover that grows from 300 to 600 mm in height. It has green, oval-shaped leaves with wounded base occurring in opposite pairs. The flower is white-cream colored with purple markings and the fruit is a club-shaped capsule, splitting from tip to base.[8,9] It is widely distributed in the tropics, including Nigeria.[10] The leaves have been shown to contain high amounts of proteins, amino acids, minerals, sugars, lipids and fiber,[11] and it is consumed as vegetable in the Eastern part of Nigeria.[12]

Its decoction has been reported to be used, traditionally, in the treatment of stomach pain, stomach worms and migraine.[9] The leaves are also claimed to be highly effective in the local treatment of asthma, and this has been validated by Akah *et al*.[9] and Ezike *et al*.,[13] who also reported the phytochemicals present. As there are no empirical data or scientific investigations on the effects of this herb on diabetes mellitus, this research was aimed at looking into the influence of *A. gangetica* juice on alloxan-induced diabetes in rats.

## MATERIALS AND METHODS

### Plant material

Fresh leaves of *A. gangetica* were collected from the University of Agriculture Abeokuta, Ogun State, Nigeria, between 7 am and 8 am. The leaves were crushed carefully with a stainless steel mortal and squeezed by means of a fine cloth to separate the juice. The required percentage of the juice was prepared by diluting the 100% juice with distilled water. The plants were identified by carrying out macroscopical examination on plant samples as stipulated by Dalziel.[14]

### Animal experiments

Thirty healthy adult albino rats weighing between 100 g and 150 g were purchased from the Nigeria Institute of Medical Research, Yaba, Lagos, Nigeria. The rats were housed individually, partly restricted in metabolic cages to mimic a sedentary lifestyle, and food was given freely, except for the fasting period prior to blood glucose determination.

### Induction of diabetes

Diabetes mellitus was induced in overnight-fasted rats by a single intraperitoneal injection of alloxan monohydrate (150 mg/kg of body weight) dissolved in normal saline. The animals to be used as controls received normal saline. Hyperglycemia was confirmed by the elevated glucose levels in plasma, determined at 72 h after injection. Animals with a blood glucose concentration of >190 mg/dl were used for the study.

### Experimental design

In the experiment, the rats were divided into six groups of five rats each as follows:

Group I (normal rats), Group II (diabetic control, receiving distilled water), Group III (diabetic, receiving glibeclamide as a standard oral antidiabetic agent at a dose of 0.45 mg/kg body weight daily), Group IV (diabetic, receiving 25% juice at a dose of 5 ml/kg body weight of rat daily), Group V (diabetic, receiving 50% juice at a dose of 5 ml/kg body weight of rat daily) and Group VI (diabetic, receiving 75% juice at a dose of 5 ml/kg body weight of rat daily). All administrations were carried out between 7 am and 9 am daily.

The blood samples for the determination of initial (after induction of diabetes) glucose concentrations were collected from the tail of the rats after they were fasted overnight. After 5 weeks of treatment, the rats were sacrificed and blood was collected by cardiac puncture into a tube containing fluoride oxalate as anticoagulant. The blood samples were collected between 7 am and 9 am after the rats were fasted overnight. The plasma was separated from the erythrocyte by centrifuging the whole blood at 250 g for 10 min.

### Analytical procedure

#### Glucose assay

The glucose assay was performed based on the colorimetric enzyme method (Cromatest^®^ Diagnostics, Joaquim Costal, Montgat, Barcelona, Spain). In this method, glucose, with the effect of glucose oxidase, can be enzymatically oxidized to gluconic acid and hydrogen peroxide. In the presence of peroxidase, the hydrogen peroxide reacts with 4-amino antipyrine and N-ethyl-N-sulfopropyl-m-toluidine to form violet-colored quinoneimine, which has an absorbance peak at 520 nm.[15]

#### Lipid profile assay

Triacylglycerol,[16] total cholesterol,[17] and high-density lipoprotein (HDL)-cholesterol[17] and HDL-triglyceride[16] were determined by colorimetric methods after enzymatic reaction with peroxidase (Cromatest^®^ Diagnostics). HDL was isolated by precipitating VLDL and low-density lipoprotein (LDL) cholesterol with heparin-manganese chloride solution.[18] LDL cholesterol was estimated by the Friedewald formula,[19] which is reliable when triacylglycerol levels are lower than 400 mg/dl.

#### Bicarbonate assay

Bicarbonate was assayed enzymatically as the level of HCO_3_ - in the plasma as described by Forrester *et al*.,[20] using a Sigma diagnostic kit (Sigma Chemical Company, St. Louis, MO, USA). In this assay, 3.0 ml of reconstituted substrate (1.79 mmol of phosphoenolpynuvate, 0.35 mmol of NADH, 212 U of phosphoenolpyruvate carboxylase and 1,250 U of malate dehydrogenase) is pipetted into a cuvette and is placed in a spectrophotometer to equilibrate at 30oC for 5 min. Ten microliters of plasma sample was added and the change in absorbance at 340 nm was read over 5 min. The amount of bicarbonate in mmol/l was calculated using 6.22, the millimolar absorptivity of NADH at 340 nm.

#### Thiobarbituric acid reactive substances (TBARS) assay

TBARS was determined colorimetrically by the method of Buege and Aust.[21] To 0.5 ml plasma, 1 ml of trichloroacetic acid was added and the tube was left to stand for 10 min at room temperature. After centrifugation at 3,500 rpm for 10 min, the supernatant was separated. Thereafter, 0.5 ml of the supernatant was added to 0.5 ml of thiobarbituric acid (TBA) and incubated in a boiling water bath for 30 min. After cooling in cold water, the resulting chromogen absorbance was determined at the wavelength of 532 nm.

### Statistical analysis

Data analyses were performed using SPSS software (SPSS 15.0 for Windows, SPSS Inc., Chicago, IL, USA). All data are expressed as mean ± SEM. Analysis of variance was used to test for differences between the groups. Duncan’s multiple range test was used to determine the significance of differences among the mean values at the level of P <0.05.

## RESULT

Body weight and plasma glucose at the beginning and end of the treatment are shown in Table 1. The juice of *A. gangetica* leaves, at all the three dosages, suppress the rise in blood glucose concentration to a level not significantly different (*P* < 0.05) from the glibenclamide-treated group and control. However, the diabetic-untreated group had a significantly (*P* < 0.05) reduced weight and increased blood glucose.

**Table 1:** Effects of *A. gangetica* juice on body weight and plasma glucose

Groups	Body weight (g)		Plasma glucose (mg/dl)	
	Initial	Final	Initial	Final
Normal control	106.25 ± 6.25^a^	112.50 ± 7.22^b^	80.25 ± 2.50^a^	76.05 ± 1.52^a^
Diabetic control	106.25 ± 11.98^a^	95.25 ± 3.42^a^	197.56 ± 2.80^b^	182.25 ± 11.25^b^
Diabetic + glibenclamide	106.25 ± 6.27^a^	143.75 ± 6.97^c^	193.00 ± 2.95^b^	73.82 ± 4.36^a^
Diabetic + 25% *A. gangetica* juice	108.75 ± 11.97^a^	101.25 ± 2.06^b^	197.68 ± 2.54^b^	81.75 ± 3.61^a^
Diabetic + 50% *A. gangetica* juice	105.50 ± 11.36^a^	115.75 ± 0.24^b^	201.36 ± 2.60^b^	71.67 ± 2.35^a^
Diabetic + 75% *A. gangetica* juice	108.33 ± 5.90^a^	125.00 ± 0.24^b^	197.58 ± 2.38^b^	79.58 ± 3.35^a^

Values are expressed as mean ± SEM. Values within the same column with different superscripts are significantly different at *P* <0.05

**Figure 1:**
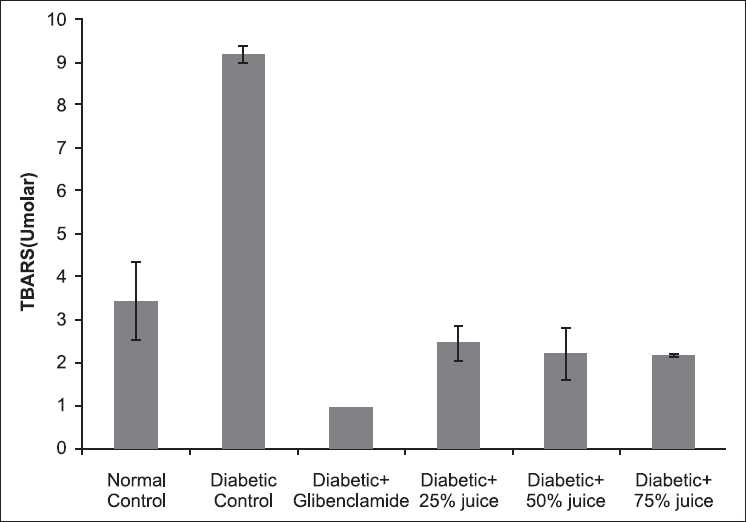
Effect of *A. gangetica* juice on TBARS level in plasma of the experimental rats (bars represent mean ± SEM)

As illustrated in Figure 1,*A. gangetica* juice and glibenclamide significantly (*P* < 0.05) suppressed the concentration of TBARS in the plasma of the diabetic rats, with glibenclamide giving the least value.

In Table 2, the levels of total cholesterol and LDL cholesterol were significantly (*P* < 0.05) higher in the diabetic control than in the normal control and in all treated groups. The three doses of *A. gangetica* juice showed no significant difference (*P* > 0.05) when compared with the nondiabetic control and the glibenclamide-treated group.

**Table 2:** Effects of *A. gangetica* juice on the plasma lipid profile of the experimental rats

Groups	Total cholesterol (mg/dl)	Triglyceride (mg/dl)	HDL-cholesterol (mg/dl)	LDL-cholesterol (mg/dl)
Normal control	64.25 ± 3.28^a^	72.00 ± 15.77^a^	46.38 ± 7.46^a^	42.28 ± 11.07^a^
Diabetic control	256.50 ± 27.25^b^	130.75 ± 19.38^b^	35.97 ± 2.59^a^	252.03 ± 29.87^b^
Diabetic + glibenclamide	66.82 ± 15.92^a^	54.09 ± 11.09^a^	42.09 ± 13.62^a^	35.54 ± 6.30^a^
Diabetic + 25% juice	55.00 ± 11.57^a^	64.25 ± 17.76^a^	118.50 ± 4.57^b^	50.65 ± 7.06^a^
Diabetic + 50% juice	41.00 ± 7.67^a^	72.50 ± 8.39^a^	109.10 ± 13.03^b^	53.60 ± 14.79^a^
Diabetic + 75% juice	38.87 ± 1.55^a^	119.33 ± 9.68^b^	104.77 ± 1.55^b^	44.23 ± 14.05^a^

Values are expressed as mean ± SEM. Values within the same column with different superscripts are significantly different at *P* <0.05

**Figure 2:**
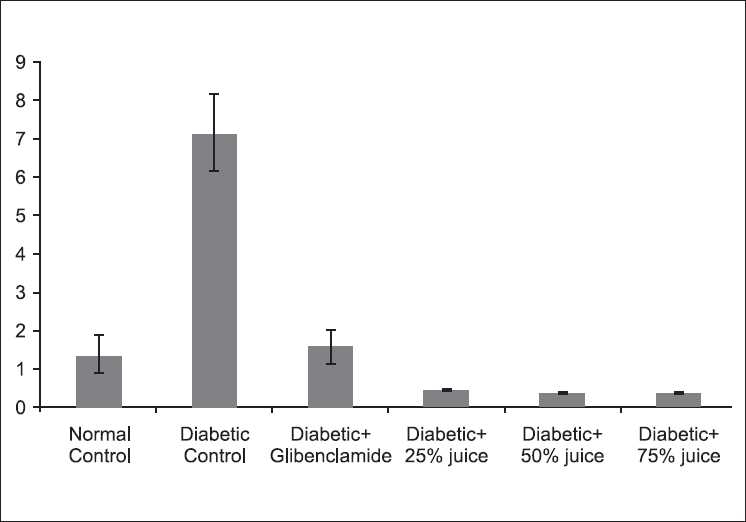
Effect of *A. gangetica* juice on total cholesterol:HDLcholesterol ratio in the experimental rats (bars represent mean ± SEM)

Figure 2 depicts the ratio of plasma total cholesterol:HDL-cholesterol in the experimental animals. The diabetic control group has the highest value, which was over 300% higher than that of the normal control. Glibenclamide returned this ratio to a level not signifcantly (*P* > 0.05) different from that of the normal control, but significantly (*P* < 0.05) higher than all the groups treated with *A. gangetica* juice.

**Figure 3:**
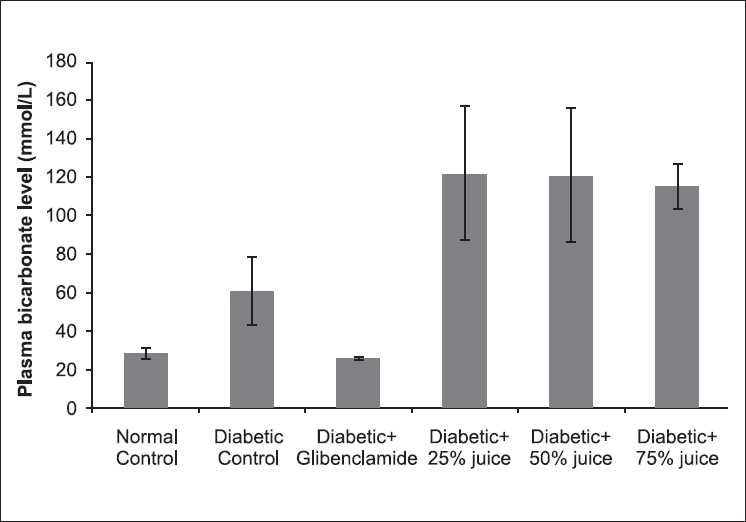
Effect of *A. gangetica* juice on plasma bicarbonate level in the experimental rats (bars represent mean ± SEM)

The influence of the treatments on plasma bicarbonate concentration was represented in Figure 3. *A. gangetica* juice lead to a further significant (*P* < 0.05) increase in plasma bicarbonate in the diabetic rats.

## DISCUSSION

The rise in blood glucose was accompanied by reduction in body weight, an observation that has been reported in other studies.[4,22] In Lee *et al*.’s[22] study, phytic acid was reported to reverse the diabetic-induced wasting. Hence, phytic acid, which is found in very high concentration in vegetables, may be responsible for the hypoglycemic effect of *A. gangetica* juice. It is well recognized that gastrointestinal autonomic neuropathy associated with disordered gastrointestinal motor and sensory function occurs frequently in diabetes.[23] This may contribute to a decrease in the food intake and an eventual weight loss, as observed in this study.

Hyperglycemia induces both oxidative stress (glucose autooxidation and advanced glycosylation end products) and a reductive stress through pseudohypoxia with NAD(P)H in the intima.[22] This redox stress overwhelms the endogenous antioxidants and initiates an oxidative destruction of membrane lipids, leading to increase in TBARS. This indirect biomarker of oxidative stress has been reported to be consistently high in diabetes.[4] The findings of this study show that *A. gangetica* juice as much as glibenclamide reversed the hyperglycemia-induced lipid peroxidation.

The mechanism by which vegetable juice exerts a protective effect appears to be related to a variety of bioactive compounds that can reduce oxidative stress.[12,24] Vegetable juice contains many antioxidants, including carotenoids, tocopherols, ascorbic acid and polyphenols, which is able to quench reactive oxidants (free radicals) and reduce oxidative damage to cell structures. Ezike *et al*.[13] reported the presence of flavonoid in *A. gangetica*. Flavonoids, apart from being antioxidants, have been reported to inhibit sodium-dependent vitamin C transporter 1 (SVCT1) and glucose transporter isoform 2 (GLUT2), the intestinal transporters for vitamin C and glucose, leading to a decrease in the intestinal absorption of glucose.[25]

Hyperglycemia and dyslipidemia as well as oxidative stress generally coexist in diabetic subjects. Dyslipidemia, which includes not only quantitative but also qualitative abnormalities of lipoproteins, plays a significant role in the proatherogenesis of vascular complications in diabetes.[2,26] It is worth nothing that *A. gangetica* juice reduced dyslipidemia and the risk of cardiovascular disease, as indicated by the reduced level of total cholesterol:HDL-cholesterol ratio. In man at least, this ratio is considered to increase the risk of coronary heart disease.[27]

Many dietary factors have been reported to contribute to the ability of herbs to improve dyslipidemia.[28] Saponin, among other secondary metabolites, is reported to be present in the leaves of *A. gangetica*.[9] This may be responsible for the lipid-lowering effect of this juice on blood lipid. Saponins may lower cholesterol by binding with cholesterol in the intestinal lumen, preventing its absorption, and/or by binding with bile acids, causing a reduction in the enterohepatic circulation of bile acids and increase in its fecal excretion.[28] Increase bile acid excretion is offset by enhanced bile acid synthesis from cholesterol in the liver and consequent lowering of the plasma cholesterol.[26] Kumarappan *et al*.[29] reported that administration of polyphenol to alloxan-induced diabetic rats reversed hyperlipidemia, and they attributed this to a reduction in the activity of hepatic HMG-CoA reductase. The antidyslipidemic effect of *A. gangetica* juice can therefore be linked to the synergistic action of these phytochemicals.

Diabetes-induced hyperlipidemia is attributable to excess mobilization of fat from the adipose due to underutilization of glucose.[28] The lack of insulin and elevations of the counter-regulatory hormones lead to activation of enzymes that stimulate lipolysis in the adipose tissue and ketogenesis in the liver. During the process of ketogenesis, free fatty acids are transformed into acetoacetate and β-hydroxybutyrate. Acetone, the least-abundant ketone body, is generated by spontaneous decarboxylation of acetoacetate. Diabetic ketoacidosis is characterized by elevated ketone bodies in the blood and metabolic acidosis, as characterized by a high level of bicarbonate.[28,30] Glibenclamide, a sulphonylurea, produces hypoglycaemia by increasing the secretion of insulin from pancreas.[31] Therefore, the inability of the juice to reduce ketoacidosis suggests that the reduction of plasma glucose might be a consequence of reduced intestinal absorption rather than an increase in the uptake and/or utilization by glucose-sensitive tissues due to an increased insulin secretion from β-cells of islets of Langerhans or because of the insulin-like actions of some components of the herb. Hence, the increase in diabetic acidosis in the diabetic rats is a point of concern in using the juice of *A. gangetica* to manage diabetes.

## References

[1] King H, Aubert RE, Herman WH (1998). Global burden of diabetes, 1995-2025; Prevalence, numerical estimates and projections. Diabetes Care.

[2] Sobngwi E, Mauvais-Jarvis F, Vexiau P, Gautier JF (2001). Diabetes in Africans: epidemiology and clinical specificities. Diabetes Metab (Paris).

[3] Rotimi CN, Dunson GM, Berg K, Akinsete O, Amoah A, Owusu A (2001). In search of susceptibility Genes for Type 2 diabetes in West Africa: The design and results of the first phase of the AADM study. Ann Epidemiol.

[4] Rotimi SO, Olayiwola I, Ademuyiwa O, Adamson I (2010). Inability of legumes to reverse diabetic-induced nephropathy in rats despite improvement in blood glucose and antioxidant status. J Med Food.

[5] Ajgaonkar SS (1979). Herbal drugs in treatment of diabetes mellitus: a review. Int Diabetes Fed Bull.

[6] Marles RJ, Fanis-Worth WR (1994). Antidiabetic plants and their active constituents. Phytomedicine.

[7] Gill LS (1992). Ethnomediacal uses of plants in Nigeria.

[8] Marim R (2006). Ethnobotanical investigation among tribes in Madurai district of Tamil Nadu (India). J Ethnobiol Ethnomed.

[9] Akah PA, Ezike AC, Nwafor SV, Okoli CO, Enwerem NW (2003). Evaluation of anti-asthmatic property of *Asystasia gangetica* leaf extracts. J Ethnopharm.

[10] Soladoye MO, Sonibare MA, Nadi AO, Alabi DA (2002). Indigenous angiosperm biodiversity of Olabisi Onabanjo University permanent site. Afr J Biotech.

[11] Yeoh HH, Wong PF (1993). Food value of lesser utilized tropical plants. Food Chem.

[12] Okudu HO (2008). Effect of drying on the nutrient contents of some Nigerian green leafy vegetable. Nig J Nutri Sci.

[13] Ezike AC, Akah PA, Okoli CO (2008). Bronchospasmolytic activity of the extract and fractions of *Asystasia gangetica* leaves. Int J App Res Nat Prod.

[14] Daziel JM (1968). The useful Plants of West Tropical Africa.

[15] Trinder P (1969). Determination of glucose in blood using glucose oxidase with an alternative oxygen acceptor. Ann Clin Biochem.

[16] Biggs HG, Erikson TA, Moorehead WR (1975). A manual colorimetric assay of triglyceride in serum. Clin Chem.

[17] Zlatkis A, Zak B, Boyle AJ (1953). A new method for the direct determination of serum cholesterol. J Lab Clin Med.

[18] Gidez LI, Miller GJ, Burstein M, Slagle S, Eder HA (1982). Separation and quantitation of subclasses of human plasma high density lipoproteins by simple precipitation procedure. J Lipid Res.

[19] Friedewald WT, Levy RE, Frederickson DS (1972). Estimation on the concentration of low density lipoprotein cholesterol in plasma without use of the ultracentrifuge. Clin Chem.

[20] Forrester RL, Wataji LJ, Silverman DA, Pierre KJ (1976). Enzymatic method for the determination of CO2 in serum. Clin Chem.

[21] Buege JA, Aust SD (1978). Microsomal lipid peroxidation. Methods Enzymol.

[22] Lee S, Park H, Cho A, Jung H, Cho S, Cho Y (2005). Effects of dietary phytic acid on serum and hepatic lipid levels in diabetic KK mice. Nutr Res.

[23] Schmidt RE (2002). Neuropathology and pathogenesis of diabetic autonomic neuropathy. Int Rev Neurobiol.

[24] Kawashima A, Madarame T, Koike H, Komatsu Y, Wise JA (2007). Four week supplementation with mixed fruit and vegetable juice concentrates increased protective serum antioxidants and folate and decreased plasma homocysteine in Japanese subjects. Asian Pac J Clin Nutr.

[25] Song J, Kwon O, Chen S, Daruwala R, Eck P, Park JB (2002). Flavonoid Inhibition of Sodium-dependent Vitamin C Transporter 1 (SVCT1) and Glucose Transporter Isoform 2 (GLUT2), intestinal transporters for vitamin c and glucose. JBC.

[26] Beckman JA, Creager MA, Libby P (2002). Diabetes and atherosclerosis: epidemiology, pathophysiology and management. JAMA.

[27] Ademuyiwa O, Ugbaja RN, Rotimi SO (2008). Plasma lipid profile, atherogenic and coronary risk indices in some residents of Abeokuta in south-western Nigeria. Biokemistri.

[28] Nimenibo-Uadia R (2003). Effect of aqueous extract of Canavalia ensiformis seeds on hyperlipidaemic and hyperketonaemia in alloxan-induced diabetic rats. Biokemistri.

[29] Kumarappan CT, Rao TN, Mandal SC (2007). Polyphenolic extract of *Ichnocarpus frutescens* modifies hyperlipidemia status in diabetic rats. J Cell Mol Biol.

[30] Sefedini E, Prašek M, Metelko Ž, Novak B, Pinter Z (2008). Use of capillary β-hydroxybutyrate for the diagnosis of diabetic ketoacidosis at emergency room: our one-year experience. Diabetologia Croatica.

[31] Singh M, Balamurugan M, Gupta A, Yadav S, Sharma A, Acharya AY (2007). Antidiabetic activity of glibenclamide loaded liposomes in alloxan induced diabetic. Ars Pharm.

